# Bibliometric analysis of nicotinic acetylcholine receptors channel research (2000-2020)

**DOI:** 10.1080/19336950.2021.1882113

**Published:** 2021-02-22

**Authors:** Xueping Zhu, Yan Zhou, Guozhen Yuan, Jingjing Shi, Shuai Shi, Limei Zhang, Ruoning Chai, Yihang Du, Chenglin Duan, Yuanhui Hu

**Affiliations:** a Cardiovascular Department, Guanganmen Hospital, China Academy of Chinese Medical Sciences, Beijing, China; b Cardiovascular Department, Graduate School of Beijing University of Chinese Medicine, Beijing, China

**Keywords:** Nicotinic, acetylcholine receptor channel, citespace, vosviewer, visual analysis

## Abstract

To explore the research status, hotspots, and trends in research on nicotinic acetylcholine receptor (nAChR) channel. The Web of Science core collection database from 2000 to 2020 was used as the data source. The visual analysis software VOSviewer1.6.16 and Citespace5.7 R3 were used to visualize the studies of the nAChR channel. The national/institutional distribution, journal distribution, authors, and related research were discussed. A total of 5,794 articles were obtained. The USA and the Utah System of Higher Education were the most productive country and institution for nAChR channel research. *Journal of Biological Chemistry* was the most productive journal (212) and the most productive researcher was McIntosh, J. Michael. The first highly co-cited article was “Refined structure of the nicotinic acetylcholine receptor at 4A resolution.” The most researched area was neurosciences neurology. The hot spots of nAChR channel research were “subunit and structure of nAChR,” “activation/agonist of nAChR channel,” and “Changes in nAChRs With Alzheimer’s Disease.” The top three research frontiers of nAChR channel research were “neuropathic pain,” “neuroinflammation,” and “α7 nACHR.” The study provides a perspective to visualize and analyze hotspots and emerging trends in the nAChR channel.

There are two types of acetylcholine receptors (AChR): nicotinic AChR (nAChR) and muscarinic AChR. nAChRs are fast cationic channels, initially discovered in muscles and autonomic ganglia function, while muscarinic AChRs are class I heptahelical G-protein-coupled receptors with a slower signaling pace [1,2].

nAChRs are members of the Cys-loop ligand-gated ion channel superfamily, which also comprises the γ-aminobutyric acid type A (GABAA), glycine (Gly), and serotonin type 3 (5-HT3) receptors. nAChR is a pentamer with five homomeric or heteromeric subunits. The diversity of nAChR subunit combinations determines their ligand-binding sites, which regulate various physiological processes. For example, nAChRs can affect the cation permeability, from monovalent Na+and K+ ions to divalent Ca2+ ions. In turn, Ca2+ ions influence signal transduction, which may affect their modulation by external Ca2+ and Zn2+ cations [3–5].

Aberrant expression or activation of nAChRs cause human diseases, including addiction, schizophrenia [6], epilepsy [7], Alzheimer’s disease [8], Parkinson’s disease [9], myasthenia gravis [10], and neuropathic pain [11], making nAChRs a major neurotherapeutic target.

Bibliometric analysis has been widely used to calculate the productivity of countries, institutions, authors, and the frequency of keywords to explore research hotspots/frontiers in specific fields [12–14]. Although nAChR channels have been a hotspot of multidisciplinary research for decades, no bibliometric studies regarding the trends in nAChR channels research activity have been published. Here, we collected scientific publications on nAChR channels research in the past 21 years, then used CiteSpace and VOSviewer for data analysis and visualization to provide researchers with some direction regarding nAChR channels research [15,16].

## Data collection

**Figure 1.**
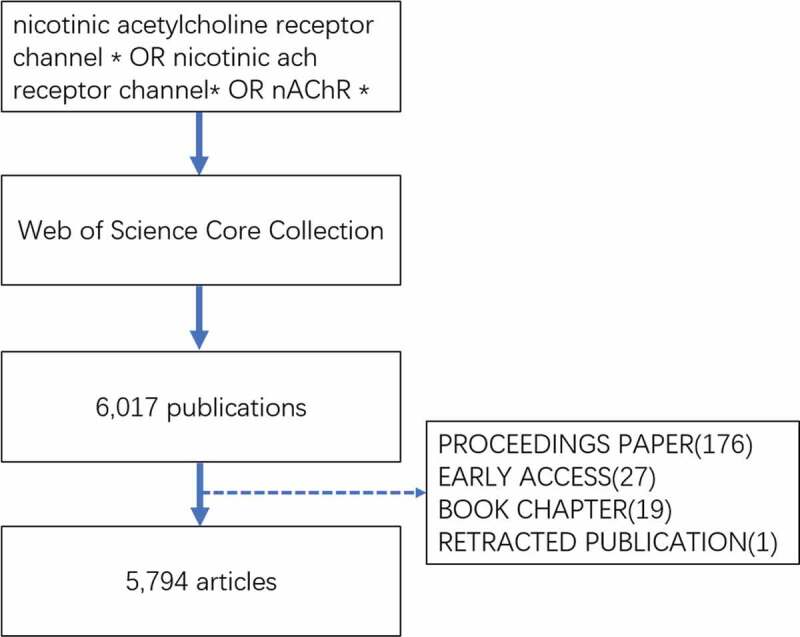
Flow chart of nAChR channels researches inclusion

The data search was conducted on 1 December 2020. The search keywords entered into the database were as follows: TS = (nicotinic acetylcholine receptor channel * OR nicotinic ACh receptor channel* OR nAChR *), language: (English) and year range: (2000–2020). The data were extracted from the Science Citation Index Expanded (SCI-expanded) of Web of Science Core Collection (WoSCC) bibliographic database, and the data were collected within 1 day to avoid any potential deviation due to the daily updating of the database. In this study, the data were downloaded directly from the database as secondary data without further animal experiments. Therefore, no ethical approval was required. Six thousand seventeen publications were obtained, and the following documents were excluded: proceedings paper (176), early access (27), book chapter (19), and retracted publication (1). In total, 5,794 articles were analyzed. The retrieval strategy of the experiments is shown in Figure 1. The VOSviewer 1.6.16 was used to identify top countries, institutions, authors, and journals. The CiteSpace 5.7 R3 was used to analyze keywords, co-cited references, and trends.

## General information and annual publication output

**Figure 2.**
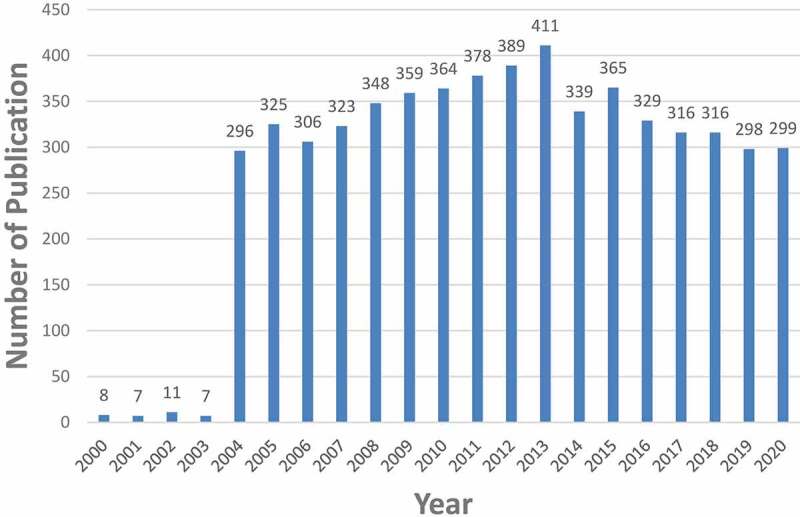
The number of annual publications on nAChR channel research from 2000 to 2020

Five thousand seven hundred and ninety-four articles were obtained. To explore the trends in nAChR channel research, we showed the number of articles per year in the form of a histogram. As shown in Figure 2, the number of publications on nAChR channel research increased gradually since 2004, reached a peak in 2013, and then began to decline, but it was still above 250. The average annual number of publications was 275.9.

## Active countries and institutions

**Figure 3.**
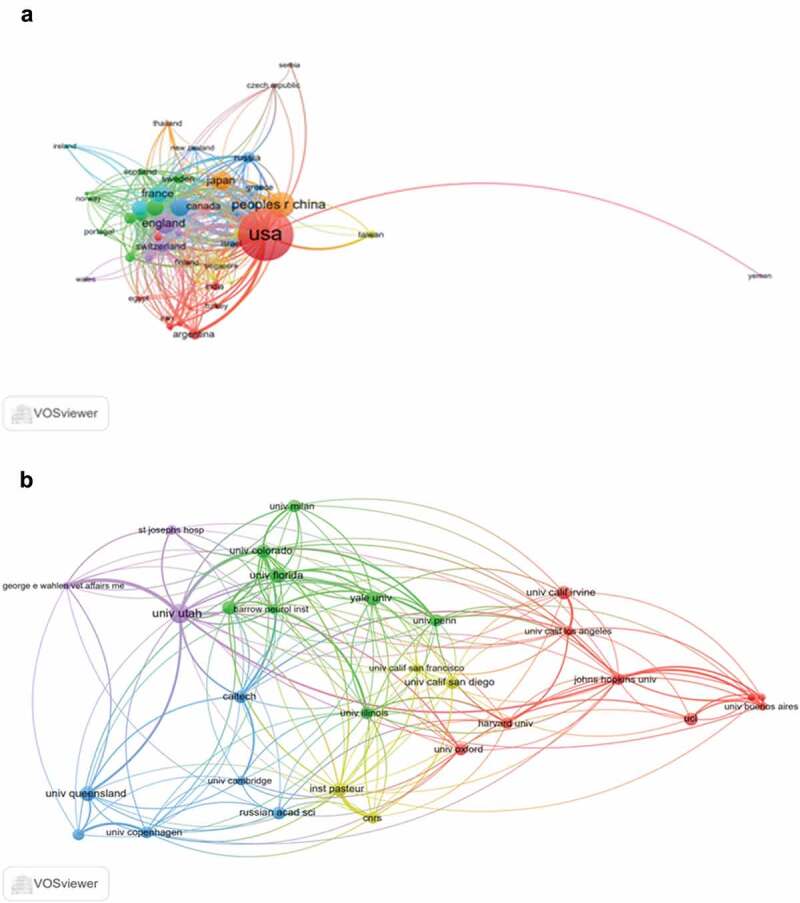
The analysis of countries and institutions. (a). The network of countries/territories engaged in nAChR channel research; (b). The network of institutions engaged in nAChR channel research

The co-occurrence map provides valuable information and helps researchers to identify the cooperative relationship [17,18]. Table 1 lists the top 10 countries and institutions that contributed to publications on the nAChR channel. Countries and institutions co-occurrence maps are shown in Figure 3 (A, B).

**Table 1.** The top 10 countries and institutions contributed to publications on nAChR channel research

Rank	Country/Territory	Frequency	Institution	Frequency
1	USA	2905	UNIVERSITY OF CALIFORNIA SYSTEM	306
2	PEOPLES R CHINA	672	UTAH SYSTEM OF HIGHER EDUCATION	209
3	ENGLAND	418	UNIVERSITY OF UTAH	208
4	GERMANY	370	CENTER NATIONAL DE LA RECHERCHE SCIENTIFIQUE CNRS	182
5	JAPAN	359	STATE UNIVERSITY SYSTEM OF FLORIDA	163
6	FRANCE	302	NATIONAL INSTITUTES OF HEALTH NIH USA	128
7	AUSTRALIA	287	US DEPARTMENT OF VETERANS AFFAIRS	127
8	ITALY	265	UNIVERSITY OF FLORIDA	123
9	CANADA	205	PENNSYLVANIA COMMONWEALTH SYSTEM OF HIGHER EDUCATION PCSHE	120
10	SWITZERLAND	161	LE RESEAU INTERNATIONAL DES INSTITUTS PASTEUR RIIP	119

The 5,794 articles on nAChR channel research were published by more than 2,931 research institutions in 87 countries/territories. The USA, Peoples R China, England, Germany, and Japan were the top five productive countries (Table 1). The United States published the most papers (2,905 articles), followed by China (672 articles), and they were the two critical countries in nAChR channel research. Figure 3(a) shows that the United States attached great importance to cooperation and had close collaborations with China, Japan, Germany, England France Australia, and Italy. Table 1 shows that American institutions published most of the publications. The Utah System of Higher Education (University of Utah) produced the highest number of publications on nAChR channels (417), followed by the University of California System (306). The co-occurrence map of institutions showed that scientific cooperation among institutions was greatly affected by the geographical location, and there are more cooperations among institutions in the same region (Figure 3).

## Active journals

The 5,794 articles were published in 1,003 journals. Table 2 lists the top 10 journals that published articles on nAChR channel research. The Journal of Biological Chemistry had the highest number at 212 (3.65%) (IF2019 = 4.238), followed by Neuropharmacology published 186 papers (3.21%) (IF2019 = 4.431), and the Journal of Neuroscience ranked third at 167 articles (2.882%) (IF2019 = 5.673).

**Table 2.** The top 10 journals that published articles on nAChR channel research

Rank	Journal	Frequency(%)	IF 2019	Country Affiliation
1	Journal of Biological Chemistry	212(3.659%)	4.238	United State
2	Neuropharmacology	186(3.21%)	4.431	England
3	Journal of Neuroscience	167(2.882%)	5.673	United State
4	Plos One	160(2.761%)	2.74	United State
5	Molecular Pharmacology	140(2.416%)	3.664	United State
6	Journal of Pharmacology And Experimental Therapeutics	119(2.054%)	3.561	United State
7	Journal of Neurochemistry	104(1.795%)	4.066	England
8	Journal of Medicinal Chemistry	98(1.691%)	6.205	United State
9	Proceedings of The National Academy of Sciences of The United States of America	89(1.536%)	9.412	United State
10	British Journal of Pharmacology	87(1.502%)	7.73	England

## Active authors

**Figure 4.**
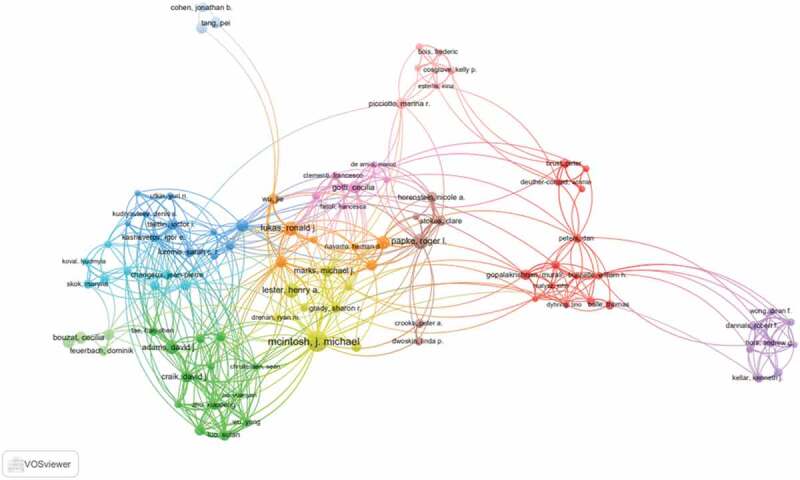
The network of authors contributed to nAChR channel research

Author co-occurrence map can provide information on influential research groups and potential collaborators. It can help researchers to find potential collaborators [17,18]. Approximately 17,830 authors contributed 5,794 articles related to nAChR channel research. The networks shown in Figure 4 indicate the cooperation among authors, and the top 10 active authors are listed in Table 3. McIntosh, J. Michael ranked first in nAChR channel publication with 160 articles, who mainly focused on alpha9 nAChRs and their role in chronic pain [19,20]. Papke, Roger L. was the second highly published author (82 articles). His research focused on the mechanisms of nAChR ligands and signaling and contributed to addiction, pain, inflammation, and other medically important issues [21–23].

**Table 3.** The top 10 active authors in nAChR channel research

Rank	Author	Frequency
1	Mcintosh JM	160
2	Papke RL	82
3	Damaj MI	75
4	Lester HA	75
5	Lukas RJ	68
6	Bertrand D	59
7	Adams DJ	57
8	Gotti C	55
9	Marks MJ	54
10	Changeux JP	52

## Co-cited references

**Figure 5.**
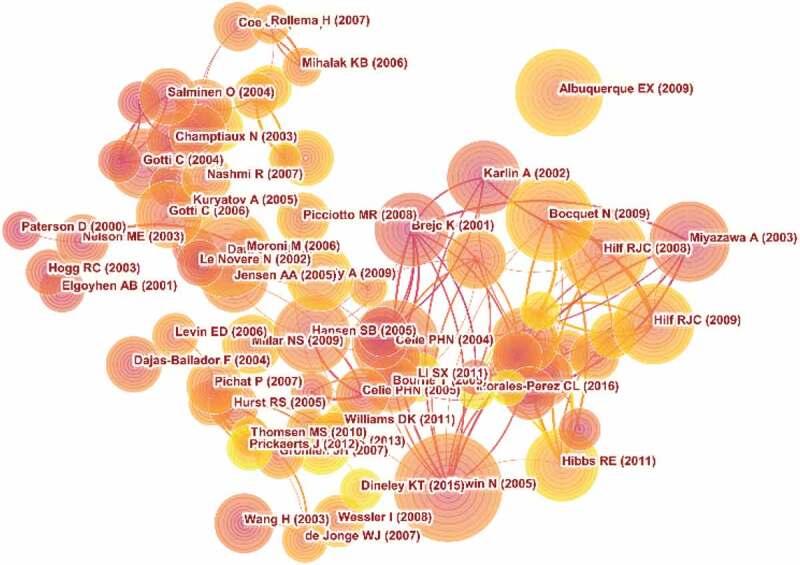
The analysis of Co-cited references: Co-citation network of references from publications on nAChR channel research

Five thousand seven hundred and ninety-four articles were visualized and analyzed using CiteSpace with a period time from 2000 to 2020, and a time slice of 1 was chosen for the analysis of the co-cited references. The network of co-cited references on nAChR channels consists of references with higher centrality and citation counts which is presented in Figure 5. The highly cited references were analyzed to determine the key knowledge base in the field. The top 10 highest co-cited references are summarized in Table 4.

**Table 4.** The top 10 Co-cited (CR) in nAChR channel research

Rank	Frequency	Author	Year	Source	Co-cited Reference
1	460	Unwin N	2005	Journal of Molecular Biology	Refined structure of the nicotinic acetylcholine receptor at 4A resolution.
2	286	Albuquerque EX	2009	Physiological Reviews	Mammalian nicotinic acetylcholine receptors: from structure to function
3	284	Brejc K	2001	Nature	Crystal structure of an ACh-binding protein reveals the ligand-binding domain of nicotinic receptors.
4	264	Miyazawa A	2003	Nature	Structure and gating mechanism of the acetylcholine receptor pore
5	260	Celie PHN	2004	Neuron	Nicotine and carbamylcholine binding to nicotinic acetylcholine receptors as studied in AChBP crystal structures.
6	239	Bocquet N	2009	Nature	X-ray structure of a pentameric ligand-gated ion channel in an apparently open conformation.
7	226	Karlin A	2002	Nature Reviews. Neuroscience	Emerging structure of the nicotinic acetylcholine receptors.
8	214	Hilf RJC	2008	Nature	X-ray structure of a prokaryotic pentameric ligand-gated ion channel.
9	214	Hansen SB	2005	The EMBO Journal	Structures of Aplysia AChBP complexes with nicotinic agonists and antagonists reveal distinctive binding interfaces and conformations.
10	193	Hibbs RE	2011	Nature	Principles of activation and permeation in an anion-selective Cys-loop receptor.

The 10 co-cited references on nAChR channel are mainly basic research from structure to function. The first highly co-cited article was “Refined structure of the nicotinic acetylcholine receptor at 4A resolution.”(460 citation rate), in which Unwin N confirmed that two ligand-binding subunits had a different extended conformation from three other subunits in the closed channel, identified several interactions on both pairs of subunit interfaces and within the subunits, and finally proposed a refined structure of the nicotinic acetylcholine Receptor at 4 A°Resolution. There were five co-cited references published in Nature: In 2001, Brejc K studied the crystal structure of the molluscan acetylcholine-binding protein (AChBP), a structural and functional homologue of the amino-terminal ligand-binding domain of a nAChR a-subunit, which assisted in the development of anti-Alzheimer’s disease and nicotine addiction drugs [24]. In 2003, Miyazawa A mainly focused on the structure and gating mechanism of the acetylcholine receptor pore, present an atomic model of the closed pore to shield the inner ring from the lipids [25]. In 2008, Hilf RJC reveals the first structure of pentameric ligand-gated ion channels (pLGICs) at 3.3 A°resolution and provides an important model system for the investigation of the general mechanisms of ion permeation and gating within the family [26]. One year later, Bocquet N presented the X-ray structure at 2.9 A°resolution of the bacterial Gloeobacter violaceus pLGICs at pH 4.6 in an apparently open conformation [27]. In 2011, Hibbs RE presented the first three-dimensional structure at 3.3 A°resolution to explain the principles of activation and permeation in an anion-selective Cys-loop receptor [28]. These articles laid the foundation for studying the structure and mechanism of nAChR channels. Albuquerque EX and Karlin A systematically reviewed nAChR channels, including nAChR subunit structure, nAChR expression, nAChR function, and relationship with disease [29,30]. And these reviews provided a theoretical basis for the study of nAChR channels.

## Research area analysis

**Figure 6.**
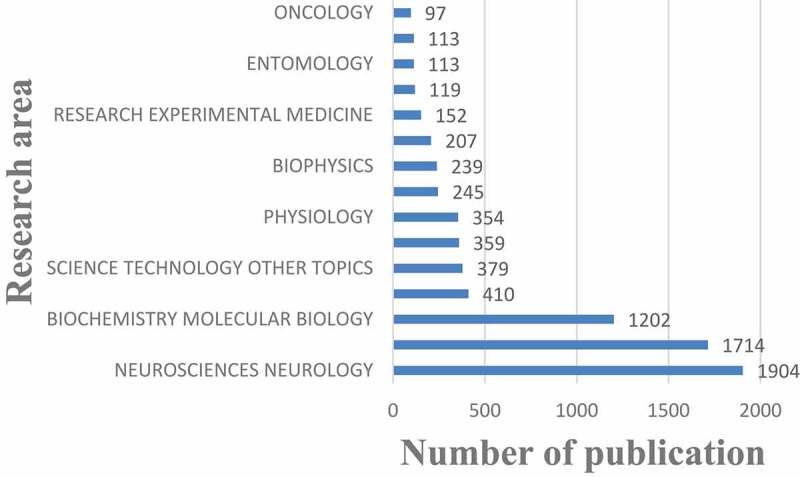
The 15 research areas on nAChR channel research

Figure 6 shows the top 15 research areas that appeared in publications related to nAChR channel research from 2000 to 2020. NEUROSCIENCES NEUROLOGY, PHARMACOLOGY PHARMACY, and BIOCHEMISTRY MOLECULAR BIOLOGY are the top three areas where nAChR channels are more studied.

## Keyword co-occurrence and burst

Keywords represent the crucial content of research. Keyword co-occurrence analysis provides a reasonable description of research hotspots, and burst keywords can represent research frontiers over a period of time [31].

**Figure 7.**
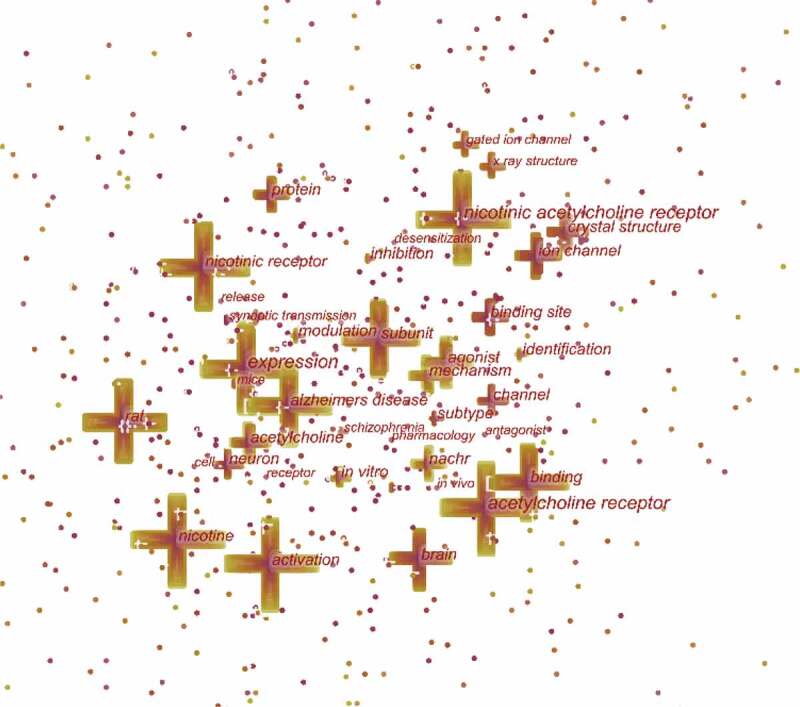
The analysis of keywords in nAChR channel research

CiteSpace 5.7.R3 was used to construct an acknowledge map of keyword co-occurrence (Figure 7) and identified the top 20 keywords in nAChR channel research articles from 2000 to 2020 (Table 5), according to frequency. The top keywords were “nicotinic acetylcholine receptor,” “acetylcholine receptor,” “expression,” “nicotine,” “activation,” “nicotinic receptor,” “subunit,” “rat,” “binding,” “Alzheimers disease,” “brain,” “agonist,” “binding site,” “ion channel,” “acetylcholine,” “crystal structure,” “mechanism,” “protein,” “neuron,” “nachr.” Therefore, research hotspots can be summarized in the following aspects:

**Table 5.** Top 20 keywords in terms of frequency in nAChR channel research

Rank	keywords	Frequency	Rank	keywords	Frequency
1	nicotinic acetylcholine receptor	1758	11	brain	376
2	acetylcholine receptor	1178	12	agonist	363
3	expression	669	13	binding site	355
4	nicotine	651	14	ion channel	340
5	activation	640	15	acetylcholine	339
6	nicotinic receptor	557	16	crystal structure	332
7	subunit	524	17	mechanism	330
8	rat	493	18	protein	321
9	binding	429	19	neuron	307
10	alzheimers disease	425	20	nachr	305

### Subunit and structure of nAChR

The AChR is an integral membrane protein that responds to the binding of acetylcholine. nAChR is a pentamer with five homomeric or heteromeric subunits, consisting of 10 α subunits (α1–10), four β subunits (β1–4), and γ, δ, and ε subunits. Although these subunits combine to form various nAChR subtypes, only two subtypes are highly expressed in the central nervous system. One is α4β2 heteromeric subunits, which have a high affinity for nicotine. Another is five α7 homomeric subunits, referred to as α7nAChR, mainly contribute to α-bungarotoxin [32,33].

The structure of nAChR subunits is composed of a large extracellular N-terminal domain (NTD; the location of the Cys-loop), three hydrophobic transmembrane regions (M1-M3), a variable intracellular loop, a fourth transmembrane region (M4), and a short extracellular C-terminus. The M1, M3, andM4 segments separate the pore-lining region from the hydrophobic membrane [2,33,34].

### Activation/agonist of nAChR channel

The cyclic C tyrosines and the cyclic B tryptophan behave similarly at all of the sites; the cyclic A tyrosine and γW55 are larger energy sources at αγ; αY190 provides most of the free energy from the affinity change in adult AChRs, but in Fetal αγ site and γW55 rank first together [35]. And in terms of agonist energy, the effects of the two agonist sites are almost independent in adult and fetal AChR because the sum of the energy measured from one site is approximately equal to the total energy of the two sites AChRs [36,37]. However, Mukhtasimova N. once proposed that there were two states before the opening of nAChR: the first state elicits brief openings, whereas the second elicits long-lived openings in 2009. And he found whether with or without agonists, long-lived openings and related activation states could be detected and showed the same dynamic characteristics [38].

### Changes in nAChRs with alzheimer’s disease

George AA demonstrated that oligomeric forms of Aβ1-42 (oAβ42) interact with α7β2-containing nAChR, and altered the intrinsic excitability of specific populations of basal forebrain cholinergic neurons (BFCNs). And he found α7β2-nAChR signaling could weaken spatial reference memory deficits in the APP/PS1 mouse model of AD [39].

Cholinesterase inhibitors, such as galantamine, are first approved to treat mild to moderately severe Alzheimer’s disease. However, the clinical potency of these drugs does not correlate well with their activity as cholinesterase inhibitors nor is their action as short-lived as purely symptomatic treatment. A subgroup of cholinesterase inhibitors can directly interact with nAChR, sensitize nicotinic receptors by increasing channel opening probability and by slowing down receptor desensitization [40].In 2008, Kowal NM evaluated galantamine actions at α4β2 and α7 nAChR in Xenopus laevis oocytes and subjected them to two-electrode voltage-clamp electrophysiological experiments. Finally, he concluded that galantamine is not a positive allosteric modulator of α7 or α4β2 receptors [41].

A new study found SK family, calcium-sensitive potassium channels, mediated inhibition exert powerful negative feedback on nicotinic excitation, dampening attention-relevant signaling in the TgCRND8 brain. These findings may contribute to a novel therapeutic target for early attention deficits in AD [42].

Keywords were identified and analyzed using strong citation bursts (Table 6) to explore the frontiers of research. As shown in Table 6, the red line indicates the period time during which the burst keyword appears [34]. The keywords that had strong bursts after 2015 were “discovery” (2015–2020), “neuropathic pain” (2015–2020), “allosteric modulation” (2016–2020), “alpha 7 nicotinic acetylcholine receptor” (2016–2020), “alpha 7 nAChR” (2017–2020), and “neuroinflammation” (2018–2020). The three research frontiers of nAChR channel research were as follows:

**Table 6.** Top 25 keywords with the strongest citation bursts

(The format of Table 6 is variable, and the picture format is as follows:)

### Neuropathic pain

Neuropathic pain is usually chronic, caused by a lesion or disorders of the peripheral or the central nervous system. And it influences 7–10% of the general population [43]. The most common conditions of neuropathic pain include trigeminal neuralgia, peripheral nerve injury, painful polyneuropathy, postherpetic neuralgia, and painful radiculopathy [44]. Studies show that neuropathic pain seriously impairs quality of life [45,46] and cause enormous economic losses to the country and society [47].

### Neuroinflammation

Neuroinflammation is common in the majority of neurological conditions. It can be both an inducement and a secondary reaction to nervous system insult [48]. The Neuroinflammation process is marked by the production of pro-inflammatory cytokines, such as IL-1β, IL-6, IL-18 and tumor necrosis factor (TNF), chemokines, small-molecule messengers, and reactive oxygen species by innate immune cells in the CNS, and microglia and astrocytes mainly involved in this process [49–51]

### α7 nACHR

The α7 nAChR is a ligand-gated ion channel and is highly expressed in the brain regions (e.g., the cerebral cortex and hippocampus) responsible for cognitive functions. The α7 nAChR is distributed both presynaptically and postsynaptically, to activate intracellular signaling cascades [32].

Burn injury (BI) pain consists of inflammatory and neuropathic components. BI significantly increased spinal cord microgliosis, microglia activation, and pain-transducer (protein and/or messenger RNA) expression. Zhou Yinhui found that GTS-21, a selective α7AChR agonist, mitigated pain-transducer changes, while the α7AChR antagonist could nullify the beneficial effects of GTS-21 [51].

Chronic cerebral hypoperfusion (CCH) induces an inflammatory response and contributes to cognitive impairment. It is demonstrated that activating of α7nAChR and its downstream JAK2-STAT3 pathway could promote cognitive function and improve neuroprotective effects against inflammation in CCH rats [52].

## Conclusions

Based on the WOSCC database, bibliometric and Visual analysis was used to study the characteristics of nAChR channel research results from 2000 to 2020. Since 2004, the number of publications on nAChR channels has maintained approximately 300 per year. The three hot spots of nAChR channel research were “subunit and structure of nAChR,” “activation/agonist of nAChR channel,” and “Changes in nAChRs With Alzheimer’s Disease.” The top three research frontiers were “neuropathic pain,” “neuroinflammation,” and “α7 nAChR.” Bibliometric analysis of the literature on the nAChR channels contributes researchers to identify cooperations, find research hotspots, and predict the frontiers of nAChR channel research.
